# MicroRNA signature constituted of miR-30d, miR-93, and miR-181b is a promising prognostic marker in primary central nervous system lymphoma

**DOI:** 10.1371/journal.pone.0210400

**Published:** 2019-01-07

**Authors:** Yasuo Takashima, Atsushi Kawaguchi, Yasuo Iwadate, Hiroaki Hondoh, Junya Fukai, Koji Kajiwara, Azusa Hayano, Ryuya Yamanaka

**Affiliations:** 1 Laboratory of Molecular Target Therapy for Cancer, Graduate School for Medical Science, Kyoto Prefectural University of Medicine, Kyoto, Japan; 2 Center for Comprehensive Community Medicine, Faculty of Medicine, Saga University, Saga, Japan; 3 Department of Neurosurgery, Graduate School of Medical Sciences, Chiba University, Chiba, Japan; 4 Departments of Neurosurgery, Toyama Prefectural Central Hospital, Toyama, Japan; 5 Department of Neurological Surgery, Wakayama Medical University School of Medicine, Wakayama, Japan; 6 Department of Neurosurgery, Graduate School of Medical Sciences, Yamaguchi University, Ube, Yamaguchi, Japan; Hokkaido Daigaku, JAPAN

## Abstract

MicroRNAs (miRNAs) are small RNA molecules that inhibit gene function by suppressing translation of target genes. However, in primary central nervous system lymphoma (PCNSL), the biological significance of miRNAs is largely unknown, although some miRNAs are known to be prognosis markers. Here, we analyzed 847 miRNAs expressed in 27 PCNSL specimens using microarray profiling and surveyed miRNA signature for prognostic prediction. Of these, 16 miRNAs were expressed in 27 PCNSL specimens at a frequency of 48%. Their variable importance measured by Random forest model revealed miR-192, miR-486, miR-28, miR-52, miR-181b, miR-194, miR-197, miR-93, miR-708, and let-7g as having positive effects; miR-29b-2*, miR-126, and miR-182 as having negative effects; and miR-18a*, miR-425, and miR-30d as neutral. After principal component analysis, the prediction formula for prognosis, consisting of the expression values of the above-mentioned miRNAs, clearly divided Kaplan-Meier survival curves by the calculated Z-score (HR = 6.4566, P = 0.0067). The 16 miRNAs were enriched by gene ontology terms including angiogenesis, cell migration and proliferation, and apoptosis, in addition to signaling pathways including TGF-β/SMAD, Notch, TNF, and MAPKinase. Their target genes included BCL2-related genes, HMGA2 oncogene, and LIN28B cancer stem cell marker. Furthermore, three miRNAs including miR-181b, miR-30d, and miR-93, selected from the 16 miRNAs, also showed comparable results for survival (HR = 8.9342, P = 0.0007), suggestive of a miRNA signature for prognostic prediction in PCNSL. These results indicate that this miRNA signature is useful for prognostic prediction in PCNSL and would help us understand target pathways for therapies in PCNSL.

## Introduction

Primary central nervous system lymphoma (PCNSL), a rare subgroup of diffuse large B-cell lymphoma (DLBCL) arising in the central nervous system (CNS), is an aggressive malignant variant of nodal non-Hodgkin lymphoma (NHL) [1,2]. PCNSLs account for 3% of all primary CNS tumors and 1% of NHLs in adults [3]. Most PCNSLs are immune privilege site-associated DLBCLs, according to the WHO diagnostic criteria [1]. Despite intensive treatments including high-dose methotrexate (HD-MTX) based polychemotherapy with whole brain radiotherapy, the median overall survival (OS) time is approximately 4 years for PCNSLs and shows a poorer prognosis than that of extracerebral DLBCLs [4–6].

MicroRNAs (miRNAs) are small noncoding regulatory RNAs consisting of approximately 20-mer nucleotides, that inhibit gene function through suppression of translation of target genes [7]. Previous studies have reported that various miRNAs are involved in cell proliferation, differentiation, cancer, and cell death in all living organisms [8,9]. The mechanism of gene silencing by miRNAs is well known and is referred to as RNA interference (RNAi) [10]. Dysregulations of miRNA expression and RNAi mechanism are related to tumor malignancy in chronic lymphocytic leukemia [11,12] and acute lymphoblastic leukemia [13]. Although molecular biology of miRNA in PCNSL is largely unknown, the expression pattern of miRNAs has been reported in PCNSL and non-CNS DLBCL [14–16]. In PCNSL, miR-199a, miR-214, miR-193b, and miR-145 were down-regulated [17]. Inversely, miRNAs that were up-regulated include miR-17-5p and miR-20a (associated with MYC pathways), miR-9 and miR-30b/c (associated with blocking of terminal B-cell differentiation), and miR-155 (associated with cytokine-dependent expression) [17,18].

Many miRNAs are known to be potential biomarkers for diagnosis and prognosis in PCNSL [19–25]. In addition, there are potential biomarker miRNAs for PCNSL in the cerebrospinal fluid [26,27] and in the serum [28]. However, to date, the comprehensive function, significance, and effectiveness of miRNAs as biomarkers in the clinical studies of PCNSL have not been elucidated.

Here, we deciphered the miRNA signature through analysis between the expression patterns of miRNAs and their correlation to the prognosis in 27 PCNSL specimens. First, we selected 16 miRNA candidates from the 847 miRNAs detected using microarray technique. Then based on principal component analysis (PCA) after Random forest analysis and clustering analysis, we determined that miR-181b, miR-30d, and miR-93 constituted a miRNA signature in PCNSL. The results here indicate that this miRNA signature is useful for prognostic prediction in PCNSL and would help us understand target pathways for therapies in PCNSL.

## Materials and methods

### Clinical specimens

Twenty-seven patients with PCNSL were diagnosed and treated at Chiba University, Toyama Prefectural Central Hospital, Wakayama Medical University School of Medicine, and Yamaguchi University. The study was approved by The Ethics Committee of Kyoto Prefectural University of Medicine (RBMR-C-1082-1), and experiments were performed in accordance with institutional guidelines. Written informed consent was obtained from all the patients.

### RNA extraction and microarray hybridization

Total RNA was extracted from approximately 100 mg of each tumor tissue using Isogen (Nippongene, Toyama, Japan). The quality of the extracted RNA was verified with a Bioanalyzer System using RNA Pico Chips (Agilent Technologies, Tokyo, Japan). Approximately 1 μg of RNA, amplified twice, was used for hybridization with Affymetrix GeneChip miRNA Array, comprising of 30,424 probes (Affymetrix, Inc., Tokyo, Japan). After hybridization, the array chips for target detection were processed, washed, and stained using the Fluidics Station 450. The High-Resolution Microarray Scanner 3000 was used for scanning the signal, and GCOS Workstation Version 1.3 was employed for image-quality analysis (Affymetrix, Inc.). The values of miRNA expression were determined using Affymetrix Expression Console Software according to manufacturer’s instructions (Affymetrix, Inc.). Arrays were normalized using a quantile normalization to impose the same empirical distribution of intensities and a Z-score was calculated as a standard deviation from their means, as described [29]. The microarray data was uploaded to the Gene Expression Omnibus (GEO) (GSE122011).

### Real-time quantitative polymerase chain reaction (qPCR)

Real-time qPCRs were performed using a StepOne Real-Time PCR System (Applied Biosystems, CA) and TaqMan MicroRNA Assays, Inventoried, SM (Applied Biosystems) according to manufacturer’s instructions. The probe/primer sets used were as follows: hsa-miR-30d (002305), hsa-miR-93 (001090), hsa-miR-181b (462578_mat), and U6 snRNA (001973) (Applied Biosystems). The values of U6 snRNA were used for normalization of expression of each miRNA.

### Gene ontology (GO) annotation

miRNAs were annotated using miRBase 22 (http://www.mirbase.org/) and their targets were surveyed with TargetScanHuman 7.2 (http://www.targetscan.org/vert_72/) on Human GRCh38/hg38 (https://genome.ucsc.edu/). Predicted targets of miRNAs with the top 30 TargetScan context++ scores [30] were listed in tables. Functional GO annotation was performed using miRBase, GOstat (http://gostat.wehi.edu.au/), and DAVID (https://david.ncifcrf.gov/), as described [31].

### Random survival forests analysis

Random survival forests analysis was used to determine the variable importance factors distinguishing expression of miRNAs with microarray raw data, as described [29,32,33]. The values of variable importance reflecting the relative contribution of each variable to the prediction for the survival time, and they were estimated by randomly permuting its values and recalculating the predictive accuracy of the model, which were expressed as the log rank test statistics. The method was implemented by using the randomForestSRC package of the statistical software R.

### Clustering analysis

Expression of miRNAs in the 27 PCNSLs was clustered with the hierarchical method using the JMP built-in modules (SAS Institute, Inc., Tokyo, Japan), as described [34].

### Kaplan-Meier survival analysis

The Kaplan-Meier method was used to estimate survival distributions for each subgroup with the log-rank test among subgroups using the JMP built-in modules (SAS Institute Inc.), as described [34].

### Statistics

Statistical analyses were performed using the JMP built-in modules (SAS Institute Inc.) as described [34]. The P-values < 0.05 were considered statistically significant.

## Results

### Patients’ characteristics

**Fig 1:**
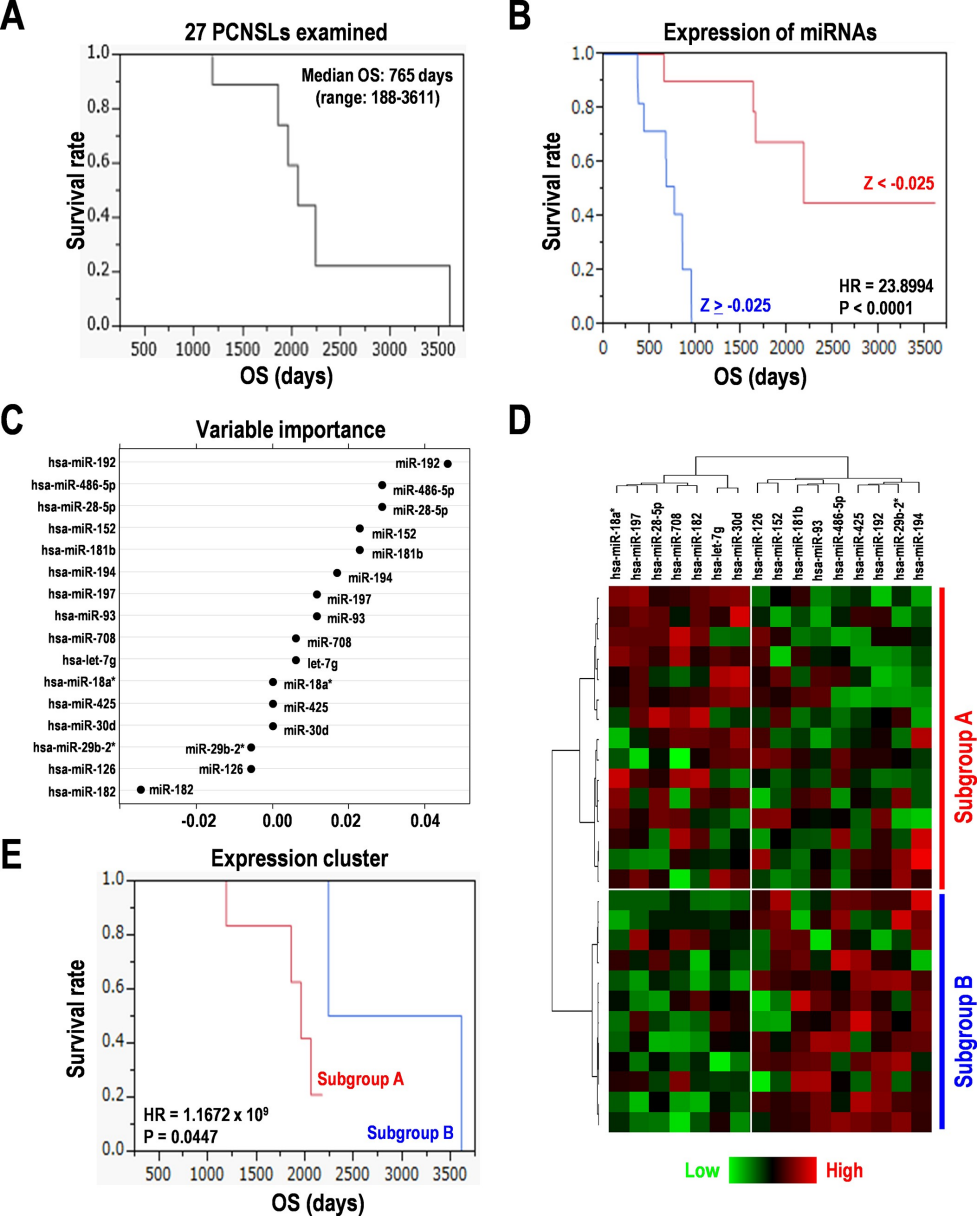
Variable importance of the 16 miRNAs with Random forest model in 27 PCNSL specimens. (**A**) Kaplan-Meier survival analysis of the 27 PCNSL specimens examined. (**B**) Survival analysis of the subgroups with higher and lower expression of miRNAs. (**C**) Variable importance of the 16 miRNAs. (**D**) Hierarchical clustering analysis from the expression of the 16 miRNAs. Red and green indicate high and low expression, respectively. (**E**) Survival analysis of the subgroups divided by the expression cluster of the 16 miRNAs.

This study was carried out on specimens from 27 PCNSL patients whose characteristics are described here (Table 1). The median age of the patients was 64 years (range, 31–76 years). Of the 27 patients, 14 patients were female (51.85%), and 13 patients were male (48.14%). The median survival time was 765 days (range, 188–3611 days) (Fig 1A), and the overall survival (OS) status was “deceased” in 14 (51.85%) and “living” in 13 patients (48.14%) at the time of collection of clinical data. The patients were treated with HD-MTX (20 patients, 74.07%) or HD-MTX-containing polychemotherapy including cyclophosphamide, pirarubicin, etoposide, vincristine, procarbazine with/without rituximab [29] (7 patients, 25.92%). Multivariate analyses for OS according to age, gender, Karnofsky Performance score (KPS), Memorial Sloan Kettering Cancer Center (MSKCC) risk score, and International Extranodal Lymphoma Study Group (IELSG) risk score [29] were performed; however, these results did not show any statistically significant difference (Table 1).

**Table 1:** Characteristics of 27 patients with PCNSL.

		OS^1^ (days)				
			Univariate		Multivariate	
Characteristics	N (%)	Median (Min—Max)	HR^2^ (95% CI)	P-value	HR (95% CI)	P-value
**Total**	**27 (100)**	**765 (188–3611)**				
**Age: Median (Min—Max)**						
** 64 (31–76)**						
** Age** **≥** **50**	**23 (85.18)**	**765 (188–3611)**	**1.4807957 (0.3151588–5.1840819**	**0.5819**	**N/A**	**-**
** Age < 50**	**4 (14.81)**	**615.5 (268–2244)**	**1**	**-**	**N/A**	**-**
**Gender**						
** Female**	**14 (51.85)**	**676 (188–2063)**	**0.4532748 (0.1166715–1.5286577)**	**0.2033**	**0.5725346 (0.1079734–2.6739615)**	**0.478**
** Male **	**13 (48.14)**	**808.5 (268–3611)**	**1**	**-**	**1**	**-**
**KPS**^**3**^**: Median (Min—Max)**						
** 70 (40–100)**						
** 0–40**	**2 (7.40)**	**374 (372–376)**	**10.195803 (0.377919–279.26015)**	**0.1425**	**5.176162 (0.1151182–232.89945)**	**0.3688**
** 50–70**	**15 (55.55)**	**950 (188–2244)**	**1.0718919 (0.3477954–3.9660423)**	**0.9079**	**1.7373379 (0.1843939–14.265166)**	**0.606**
** 80–100**	**10 (37.03)**	**718.5 (214–3611)**	**1**	**-**	**1**	**-**
**MSKCC**^**4**^						
** 1 (Age < 50)**	**4 (14.81)**	**615.5 (268–2244)**	**1**	**-**	**1**	**-**
** 2 (Age** **≥** **50, KPS** **≥** **70)**	**12 (44.44)**	**903.5 (214–3611)**	**1.4882871 (0.2484691–28.404369)**	**0.7027**	**2.05E+08 (0.1527173–3.65E+230)**	**0.2911**
** 3 (Age** **≥** **50, KPS < 70)**	**11 (40.74)**	**433 (188–2179)**	**3.2489292 (0.5672479–61.184808)**	**0.2099**	**3.52E+08 (0.3138735–4.77E+230)**	**0.1833**
**IELSG**^**5**^						
** 0–1**	**3 (11.11)**	**953 (358–2244)**	**N/A**	**-**	**N/A**	**-**
** 2–3**	**23 (85.18)**	**765 (188–3611)**	**N/A**	**-**	**N/A**	**-**
** 3–5**	**1 (3.70)**	**214 (214–214)**	**N/A**	**-**	**N/A**	**-**
**Treatment**						
** HD-MTX**^**6**^	**20 (74.07)**	**808.5 (188–3611)**	**1.0742778 (0.1159885–8.2652953)**	**0.9447**	**0.593969 (0.1557161–2.2610745)**	**0.4347**
** Polychemotherapy**	**7 (25.92)**	**676 (214–2179)**	**1**	**-**	**1**	**-**
**Z-score (Affimetrix microarray): Median (Min—Max)**						
** -0.025113 (-3.776–5.039)**						
** Z-score > -0.025**	**11 (40.74)**	**676 (363–953)**	**23.89946 (4.2468904–452.12653)**	<0.0001*	**5.9414E+09 (4.899272–7.48E+232)**	0.0024*
** Z-score < -0.025**	**16 (59.25)**	**1409.5 (188–3611)**	**1**	**-**	**1**	**-**

Note

^1^OS; overall survival

^2^HR; hazard ratio

^3^KPS; Karnofsky Performance score

^4^MSKCC; Memorial Sloan Kettering Cancer Center risk score

^5^IELSG; International Extranodal Lymphoma Study Group risk score

^6^HD-MTX; high-dose methotrexate

*P < 0.05, statistically significant, N/A; not applicable.

### MicroRNA expression in PCNSL

First, to investigate the expression of miRNAs in the 27 PCNSL specimens, we performed microarray experiment using a miRNA GeneChip comprising 30,424 probes and 847 human miRNAs were detected (S1 Fig). The subgroup with highly expressed miRNAs had high hazard ratio (HR) than those with lower expressed miRNAs (Z-score = -0.025; HR = 5.9414 × 10^9^; 95% CI = 4.8992–7.48 × 10^232^; P = 0.0024, in multivariate analysis) (Table 1 and Fig 1B), suggesting that dysregulated miRNA pathways were correlated to poor prognosis and/or their malignancies in PCNSL. After summarizing the expression data of miRNAs, we selected 16 universally expressed miRNAs that were relatively highly expressed at a frequency of 48% in all 27 PCNSLs (Table 2). Representative gene ontology (GO) terms (GO numbers) of these miRNAs were enriched by angiogenesis (0045766, 1904995, 0090050, 1903589, 1905564, 0043537, 1905563, and 1904036), proliferation (1903589, 1905564, 1905563, 0120041, and 1904706), cell migration (0090050, 0043537, 0030335, and 1904753), and apoptosis (1904036), and the signaling pathways including TGF-β/SMAD (0030512 and 0060394), Notch (0045747), TNF (0010804), and p38 MAP Kinase (1900745) (Table 3). The targets of these miRNAs included (i) cell growth-related genes namely proto-oncogene HMGA2, cancer stem cell marker LIN28B, and MAP Kinase-related genes RBAK and RGS17; (ii) BCL2-related genes BNIP2 and BAG1; (iii) ubiquitin-related genes TRIM71 and USP44; and (iv) transcription factors ARID3B, POU2F2, and NR6A1 (Table 4).

**Table 2:** Universally expressed miRNAs in 27 PCNSL specimens.

miRNA ID	Sequence	Alignments	Detection Rate
**hsa-miR-126**	**UCGUACCGUGAGUAAUAAUGCG**	**Chr9:138684875–138684959 (+)**	**1.00**
**hsa-miR-181b**	**AACAUUCAUUGCUGUCGGUGGGU**	**Chr1:197094625–197094734 (-) // Chr9:126495810–126495898 (+)**	**1.00**
**hsa-miR-182**	**UUUGGCAAUGGUAGAACUCACACU**	**Chr7:129197459–129197568 (-)**	**1.00**
**hsa-miR-28-5p**	**AAGGAGCUCACAGUCUAUUGAG**	**Chr3:189889263–189889348 (+)**	**1.00**
hsa-miR-29b-2*	**CUGGUUUCACAUGGUGGCUUAG**	**Chr1:206042411–206042491 (-) // Chr7:130212758–130212838 (-)**	**1.00**
**hsa-miR-30d**	**UGUAAACAUCCCCGACUGGAAG**	**Chr8:135886301–135886370 (-)**	**1.00**
**hsa-miR-425**	**AAUGACACGAUCACUCCCGUUGA**	**Chr3:49032585–49032671 (-)**	**1.00**
**hsa-miR-93**	**CAAAGUGCUGUUCGUGCAGGUAG**	**Chr7:99529327–99529406 (-)**	**1.00**
**hsa-let-7g**	**UGAGGUAGUAGUUUGUACAGUU**	**Chr3:52277334–52277417 (-)**	**0.96**
**hsa-miR-708**	**AAGGAGCUUACAAUCUAGCUGGG**	**Chr11:78790714–78790801 (-)**	**0.96**
**hsa-miR-486-5p**	**UCCUGUACUGAGCUGCCCCGAG**	**Chr8:41637116–41637183 (-)**	**0.85**
**hsa-miR-152**	**UCAGUGCAUGACAGAACUUGG**	**Chr17:43469526–43469612 (-)**	**0.78**
**hsa-miR-194**	**UGUAACAGCAACUCCAUGUGGA**	**Chr11:64415403–64415487 (-) // Chr1:218358122–218358206 (-)**	**0.74**
hsa-miR-18a*	**ACUGCCCUAAGUGCUCCUUCUGG**	**Chr13:90801006–90801076 (+)**	**0.70**
**hsa-miR-197**	**UUCACCACCUUCUCCACCCAGC**	**Chr1:109943038–109943112 (+)**	**0.63**
**hsa-miR-192**	**CUGACCUAUGAAUUGACAGCC**	**Chr11:64415185–64415294 (-)**	**0.48**

Note: The 16 miRNAs detected in about 50% of PCNSLs. The expression level over and under the median expression of each miRNA was referred as “expressed” and “not expressed”, respectively.

**Table 3:** GO analysis of the universally expressed miRNAs in 27 PCNSL specimens.

miRNA ID	GO term	Description
**hsa-let-7g-5p**	**GO:0030512**	**negative regulation of transforming growth factor beta receptor signaling pathway**
	**GO:0035195**	**gene silencing by miRNA**
	**GO:0045766**	**positive regulation of angiogenesis**
	**GO:0050728**	**negative regulation of inflammatory response**
	**GO:0060394**	**negative regulation of pathway-restricted SMAD protein phosphorylation**
	**GO:0071333**	**cellular response to glucose stimulus**
	**GO:1904995**	**negative regulation of leukocyte adhesion to vascular endothelial cell**
**hsa-miR-126-5p**	**GO:0035195**	**gene silencing by miRNA**
	**GO:0045747**	**positive regulation of Notch signaling pathway**
	**GO:0071499**	**cellular response to laminar fluid shear stress**
	**GO:0090050**	**positive regulation of cell migration involved in sprouting angiogenesis**
	**GO:1903589**	**positive regulation of blood vessel endothelial cell proliferation involved in sprouting angiogenesis**
	**GO:1905564**	**positive regulation of vascular endothelial cell proliferation**
**hsa-miR-152-3p**	**GO:0010804**	**negative regulation of tumor necrosis factor-mediated signaling pathway**
	**GO:0035195**	**gene silencing by miRNA**
	**GO:0043537**	**negative regulation of blood vessel endothelial cell migration**
	**GO:1904684**	**negative regulation of metalloendopeptidase activity**
	**GO:1905563**	**negative regulation of vascular endothelial cell proliferation**
**hsa-miR-181b-5p**	**GO:0005615**	**extracellular space**
	**GO:0030335**	**positive regulation of cell migration**
	**GO:0035195**	**gene silencing by miRNA**
	**GO:0035278**	**miRNA mediated inhibition of translation**
	**GO:0042116**	**macrophage activation**
	**GO:0097237**	**cellular response to toxic substance**
	**GO:0110015**	**positive regulation of elastin catabolic process**
	**GO:0120041**	**positive regulation of macrophage proliferation**
	**GO:1900745**	**positive regulation of p38MAPK cascade**
	**GO:1903231**	**mRNA binding involved in posttranscriptional gene silencing**
**hsa-miR-182-5p**	**GO:0030335**	**positive regulation of cell migration**
	**GO:0035195**	**gene silencing by miRNA**
	**GO:1904036**	**negative regulation of epithelial cell apoptotic process**
	**GO:1904706**	**negative regulation of vascular smooth muscle cell proliferation**
	**GO:1904753**	**negative regulation of vascular associated smooth muscle cell migration**
	**GO:1905175**	**negative regulation of vascular smooth muscle cell dedifferentiation**
**hsa-miR-18a-3p**	**GO:0005615**	**extracellular space**
**hsa-miR-192-5p**	**GO:0005615**	**extracellular space**
**hsa-miR-194-5p**	**GO:0005615**	**extracellular space**
**hsa-miR-197-3p**	**GO:0005615**	**extracellular space**
**hsa-miR-28-5p**	**GO:0035278**	**miRNA mediated inhibition of translation**
**hsa-miR-29b-2-5p**	**-**	**-**
**hsa-miR-30d-5p**	**GO:0005615**	**extracellular space**
**hsa-miR-425-5p**	**GO:0035195**	**gene silencing by miRNA**
**hsa-miR-486-5p**	**GO:0005615**	**extracellular space**
**hsa-miR-708-5p**	**-**	**-**
**hsa-miR-93-5p**	**GO:0035195**	**gene silencing by miRNA**
	**GO:0035195**	**gene silencing by miRNA**
	**GO:1903231**	**mRNA binding involved in posttranscriptional gene silencing**

**Note: miRBase,** http://www.mirbase.org/

**Table 4:** The predicted target genes of the universally expressed miRNAs in the 27 PCNSL specimens.

Target gene	Gene name	TargetScan Score	miRNA ID
**HMGA2**	**high mobility group AT-hook 2**	**-2.69**	**hsa-let-7g-5p**
**FIGN**	**fidgetin**	**-2.11**	**hsa-let-7g-5p**
**ARID3B**	**AT rich interactive domain 3B (BRIGHT-like)**	**-1.68**	**hsa-let-7g-5p**
**TRIM71**	**tripartite motif containing 71, E3 ubiquitin protein ligase**	**-1.66**	**hsa-let-7g-5p**
**LIN28B**	**lin-28 homolog B (C. elegans)**	**-1.58**	**hsa-let-7g-5p**
**HIST1H2AK**	**histone cluster 1, H2ak**	**-1.52**	**hsa-miR-18a-3p**
**PHLDA3**	**pleckstrin homology-like domain, family A, member 3**	**-1.34**	**hsa-miR-18a-3p**
**USP44**	**ubiquitin specific peptidase 44**	**-1.3**	**hsa-let-7g-5p**
**POU2F2**	**POU class 2 homeobox 2**	**-1.29**	**hsa-let-7g-5p**
**ATP6AP2**	**ATPase, H+ transporting, lysosomal accessory protein 2**	**-1.26**	**hsa-miR-152-3p**
**NR6A1**	**nuclear receptor subfamily 6, group A, member 1**	**-1.26**	**hsa-let-7g-5p**
**KLHL28**	**kelch-like family member 28**	**-1.23**	**hsa-miR-30d-5p**
**CCKBR**	**cholecystokinin B receptor**	**-1.21**	**hsa-miR-152-3p**
**SNX16**	**sorting nexin 16**	**-1.2**	**hsa-miR-30d-5p**
**BNIP2**	**BCL2/adenovirus E1B 19kDa interacting protein 2**	**-1.15**	**hsa-miR-194-5p**
**POC1B-GALNT4**	**POC1B-GALNT4 readthrough**	**-1.14**	**hsa-miR-181b-5p**
**OSBPL3**	**oxysterol binding protein-like 3**	**-1.13**	**hsa-miR-181b-5p**
**QKI**	**QKI, KH domain containing, RNA binding**	**-1.13**	**hsa-miR-152-3p**
**TEX22**	**testis expressed 22**	**-1.12**	**hsa-miR-18a-3p**
**ANKRA2**	**ankyrin repeat, family A (RFXANK-like), 2**	**-1.09**	**hsa-miR-30d-5p**
**RBAK**	**RB-associated KRAB zinc finger**	**-1.08**	**hsa-miR-181b-5p**
**RGS17**	**regulator of G-protein signaling 17**	**-1.08**	**hsa-miR-182-5p**
**PDCD1LG2**	**programmed cell death 1 ligand 2**	**-1.07**	**hsa-miR-93-5p**
**BAG1**	**BCL2-associated athanogene**	**-1.05**	**hsa-miR-28-5p**
**BAG1**	**BCL2-associated athanogene**	**-1.05**	**hsa-miR-708-5p**
**TMED7**	**transmembrane emp24 protein transport domain containing 7**	**-1.05**	**hsa-miR-152-3p**
**SNX8**	**sorting nexin 8**	**-1.02**	**hsa-miR-18a-3p**
**IGDCC3**	**immunoglobulin superfamily, DCC subclass, member 3**	**-1.01**	**hsa-let-7g-5p**
**PPP1R1C**	**protein phosphatase 1, regulatory (inhibitor) subunit 1C**	**-1.01**	**hsa-miR-182-5p**
**IMPG1**	**interphotoreceptor matrix proteoglycan 1**	**-1**	**hsa-miR-182-5p**

**Note: TargetScanHuman 7.2,** http://www.targetscan.org/vert_72/

### Random forest analysis and expression cluster in PCNSL

Next, to examine the contribution of the expression of 16 miRNAs to overall survivals of patients with PCNSL, we analyzed them by constructing a Random forest model. Variable importance measures indicated miR-192, miR-486-5p, miR-28-5p, miR-52, miR-181b, miR-194, miR-197, miR-93, miR-708, and let-7g as having positive effects; miR-29b-2*, miR-126, and miR-182 as having negative effects; and miR-18a*, miR-425, and miR-30d as neutral (Fig 1C). After clustered analysis of the expression patterns of these 16 miRNAs, the two subgroups with differential expression cluster of the miRNAs were divided by Kaplan-Meier survival curves (HR = 1.1672 × 10^9^, P = 0.0447) (Fig 1D and 1E). Among these miRNAs, we found that only miR-194^high^ indicated good prognosis with a statistically significant difference (HR = 1.075 × 10^26^, P = 0.0147) in Kaplan-Meier analysis (S2A Fig).

### MicroRNA signature for prognostic prediction in PCNSL

Based on the results from a Random forest model, expression clustering analysis, and additional principal component analysis (PCA), the formula for prognostic prediction in PCNSL was constituted using expression values of the 16 miRNAs, as follows: Z_1_ = - 0.34 × miR-29b-2* + 0.32 × miR-18a* - 0.18 × miR-708–0.1 × miR-486-5p + 0.29 × miR-425 + 0.15 × miR-194–0.11 × miR-126–0.27 × miR-152 + 0.01 × let-7g + 0.34 × miR-182–0.3 × miR-181b + 0.39 × miR-30d - 0.27 × miR-197 + 0.21 × miR-192 + 0.25 × miR-93–0.06 × miR-28-5p.

**Fig 2:**
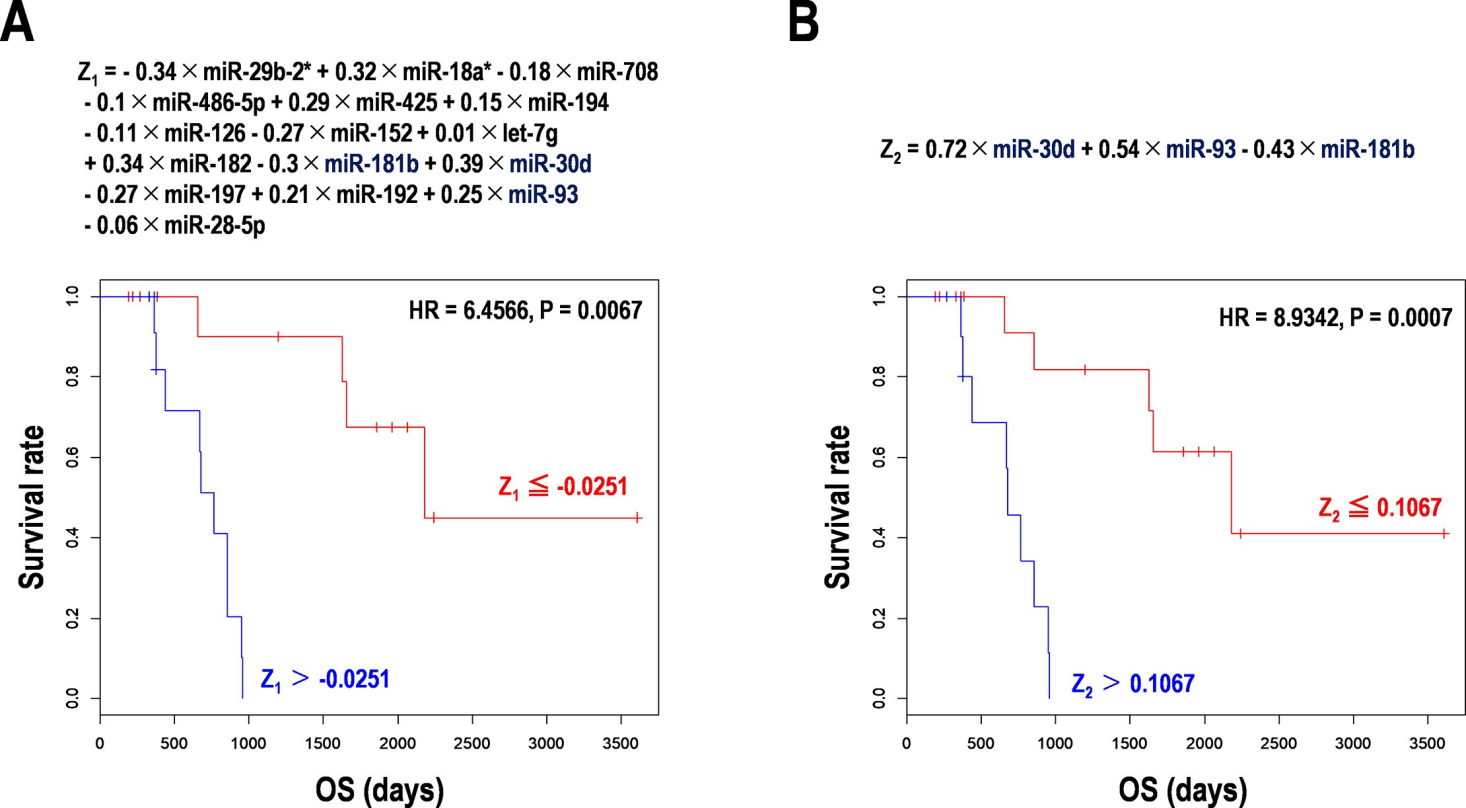
Survival prediction using miRNA signatures in 27 PCNSL specimens. Z-scores were calculated by the prediction formula with (**A**) the 16 miRNAs and (**B**) the three miRNA signatures including miR-30d, miR-93, and miR-181b. Kaplan-Meier analyses were performed. OS, overall survival (days).

The two subgroups divided by a Z_1_ score (= -0.0251) clearly divided the Kaplan-Meier curves (HR = 6.4566, 95% CI: 1.7334–24.0617, P = 0.0067) (Fig 2A). Further, the formula with reduced dimension was also reconstituted using expression values of the three selected miRNAs, as follows: Z_2_ = 0.72 × miR-30d + 0.54 × miR-93–0.43 × miR-181b.

The two subgroups divided by a Z_2_ score (= 0.1067) also extremely divided the Kaplan-Meier curves (HR = 23.45498, 95% CI: 4.448828–245.78973, P < 0.0001, in multivariate analysis) (Fig 2B and Table 5). Expression of miR-30d, miR-93, and miR-181b was validated by the qPCR method, although the correlation between the results of microarrays and qPCRs were hardly detected (S3 Fig). These results suggested that the 16 universally expressed miRNAs on the microarray function as useful prognostic markers in PCNSL, and especially, the three selected miRNAs including miR-30d, miR-93, and miR-181b are valid for the miRNA signature as a promising prognostic marker in PCNSL, whereas the exact expression of miRNAs should further be addressed in a large data set. Representative GO terms (GO numbers) of the three miRNAs were cell migration (0030335), macrophage activation (0042116) and proliferation (0120041), p38 MAPK cascade (1900745), and extracellular space (0005615) (Table 3). Besides, the targets of the three miRNAs included cell growth-related genes RBAK, GPR137C, HTR1F, and MAP3K2 (also known as MEKK2); apoptosis-related gene PDCD2L; and immune-related gene PDCD1LG2 (also known as PD-L2) (S1 Table). These results suggested that it would be a hint for development of target therapies in PCNSL.

**Table 5:** Multivariate prognostic analysis for the miRNA signature in 27 PCNSL specimens.

	Multivariate analysis for OS^1^	
Characteristics	HR^2^ (95% CI)	P-value
**Z**_**2**_**-score**		
** ** **Z**_**2**_**-score** **≥** **0.1067**	**23.45498 (4.448828–245.78973)**	<0.0001*
** ** **Z**_**2**_**-score < 0.1067**	**1**	**-**
**Age**		
** ** **Age** **≥** **50**	**0.0391494 (0.0002354–2.8069745)**	**0.1337**
** ** **Age < 50**	**1**	**-**
**KPS**^**3**^		
** ** **70 (40–100)**		
** ** **0–40**	**1.5492919 (0.0331203–64014121)**	**0.8072**
** ** **50–70**	**0.270712 (0.0219643–2.0230433)**	**0.21**
** ** **80–100**	**1**	**-**
**MSKCC**^**4**^		
** ** **1 (Age < 50)**	**N/A**	**-**
** ** **2 (Age** **≥** **50, KPS** **≥** **70)**	**N/A**	**-**
** ** **3 (Age** **≥** **50, KPS < 70)**	**N/A**	**-**
**IELSG**^**5**^		
** ** **0–1**	**N/A**	**-**
** ** **2–3**	**N/A**	**-**
** ** **3–5**	**N/A**	**-**

Note

^1^OS; overall survival

^2^HR; hazard ratio

^3^KPS; Karnofsky Performance score

^4^MSKCC; Memorial Sloan Kettering Cancer Center risk score

^5^IELSG; International Extranodal Lymphoma Study Group risk score

*P < 0.05, statistically significant, N/A; not applicable.

## Discussion

PCNSL is a rare subtype of extra-nodal NHL, in which biological significance have been poorly understood, with only a few studies on the CNS signature of PCNSL being reported compared to non-CNS DLBCL [35–37]. Previous studies have reported that, in DLBCL, the up-regulated miRNAs are miR-155, miR-210, miR-17-5p, and miR-106, whereas the down-regulated miRNAs are miR-150, miR-145, miR-328, miR-139, miR-99a, miR-10a, miR-95, miR-149, miR-320, miR-151, and let-7e, compared to that in normal lymph nodes [38]. Reduced expression of miR-195 and let-7g also shows good EFS in DLBCL [38]. In this study as well, only the down-regulated miR-10a showed poorer survival in PCNSL (S2B Fig). There is a bias in miRNA expression pattern between PCNSL and nodal DLBCL; miR-9, miR-20a/b, miR-155, miR-340, miR-17-5p, miR-148a, miR-30b/c, miR-27b, miR-26b, miR-146b, and let-7g are expressed in PCNSL; whereas miR-199a, miR-214, miR-432, miR-193b, and miR-145 are expressed in nodal DLBCL [17]. This study also demonstrated that a subgroup associated with higher expression of let-7g, a suppressor of LIN28B stem cell marker [39], showed poor prognosis in PCNSL tumors (Fig 1D and 1E). A recent study has revealed that increased expression of miR-30c in patients with secondary CNS lymphoma (SCNSL), compared to those with PCNSL, allows the lymphomas to engraft into CNS, by binding to the cadherin EGF LAG seven-pass G-type receptor (CELSR) 3 gene [40]. In non-CNS DLBCL, exosomal RNAs are considered biomarkers in the blood, serum, and cerebrospinal fluid (CSF) [19,41]. Such serum biomarkers include high expression of miR-15, miR-16, miR-21, miR-29c, miR-155, and miR-210, but low expression of miR-34a within the serum [28,42]. The miR-30c derived from the CSF can serve as a biomarker to distinguish PCNSL from SCNSL [40]. We also examined the expression of 33 above-mentioned miRNAs and their correlation to the OS in this study. However, no differences were found except for let-7g and miR-10a (Fig 1D and 1B, and S2 Fig). Thus, we should also address comprehensive expression profiling of miRNAs in a larger population of PCNSL, as well as a non-CNS DLBCL study [43].

In this study, we selected 16 universally expressed miRNAs from 847 miRNAs detected using microarray, and then determined miR-30d, miR-93, and miR-181b as the CNS miRNA signature for prognosis prediction. This study was carried out on specimens from 27 patients with PCNSL and their miRNA expressions data were analyzed using a Random forest model, principal component analysis, and Kaplan-Meier method. Interestingly, miR-30d targets programmed cell death 2-like (PDCD2L), autophagy related 12 (ATG12), serotonin receptor 1F, G-protein coupled (HTR1F) (S1 Table). The miR-93 targets programmed cell death 1 ligand 2 (PDCD1LG2, also known as PD-L2), which is related to cancer immunotherapy, and G-protein coupled receptor 137C (GPR137C), and mitogen-activated protein kinase kinase kinase 2 (MAP3K2, also known as MEKK2) in the RAS-MAPK signaling (S1 Table). The miR-181b targets retinoblastoma (RB)-associated KRAB zinc finger (RBAK) in the RB signaling (S1 Table). Altogether, these data propose that the signature constituted of miR-30d, miR-93, and miR-181b is involved in cell death and proliferation, and immune tolerance. Our data demonstrated that a subgroup associated with lower expression of miR-93 showed poor prognosis (Fig 1D and 1E), which could be explained by the genetic interaction between miR-93 and PDCD1LG2. Thereby, it might have acquired immune tolerance with PD-L2/PD-1 axis within the PCNSL microenvironments. In Hodgkin lymphoma, miR-30d and miR-93 are known to be up-regulated and down-regulated, respectively [44]. Furthermore, the plasma level of miR-93 is associated with higher mortality in 25 patients with lymphoma [44], which suggests that they are reliable biomarker candidates. Differentially expressed miR-30d is also predicted for CNS-DLBCL [45]. Although the data is limited by the small sample size and there is less evidence for the above-mentioned function and signaling pathways at present, it would help us to better understand biological significances of the CNS signature of miRNAs in PCNSL.

## Supporting information

S1 Table The predicted target genes of miR-30d, miR-181b, and miR-93. (DOCX)

S1 Fig Expression patterns of 847 miRNAs in 27 PCNSL specimens. Hierarchical clustering analysis was performed. Red and green indicate high and low expression, respectively.
(TIF)

S2 Fig Survival prediction with the expression values of each miRNA in 27 PCNSL specimens. The patients were divided by median expression values of (**A**) miR-194 and (**B**) miR-10a. Kaplan-Meier analyses were performed. OS, overall survival (days).
(TIF)

S3 Fig The correlation between the microarray and the quantitative PCR. (**A**) miR-30d, (**B**) miR-93, and (**C**) miR-181b. Scatter plots were shown with statistic results. Each dot represents a PCNSL specimen.
(TIF)
