# Supplementary material for: MicroRNA signature constituted of miR-30d, miR-93, and miR-181b is a promising prognostic marker in primary central nervous system lymphoma
Source: PLoS One. 2019 Jan 7;14(1):e0210400. doi: 10.1371/journal.pone.0210400 (PMC6322780; doi:10.1371/journal.pone.0210400)
Supplement: S1 Table — (DOCX) [file pone.0210400.s001.docx]

**S1 Table. The predicted target genes of miR-30d, miR-181b, and miR-93.**

|  | | | |
| --- | --- | --- | --- |
| **Target gene** | **Gene name** | **TargetScan Score** | **miRNA ID** |
| **KLHL28** | **kelch-like family member 28** | **-1.23** | **hsa-miR-30d-5p** |
| **SNX16** | **sorting nexin 16** | **-1.2** | **hsa-miR-30d-5p** |
| **POC1B-GALNT4** | **POC1B-GALNT4 readthrough** | **-1.14** | **hsa-miR-181b-5p** |
| **OSBPL3** | **oxysterol binding protein-like 3** | **-1.13** | **hsa-miR-181b-5p** |
| **ANKRA2** | **ankyrin repeat, family A (RFXANK-like), 2** | **-1.09** | **hsa-miR-30d-5p** |
| **RBAK** | **RB-associated KRAB zinc finger** | **-1.08** | **hsa-miR-181b-5p** |
| **PDCD1LG2** | **programmed cell death 1 ligand 2** | **-1.07** | **hsa-miR-93-5p** |
| **MTDH** | **metadherin** | **-0.98** | **hsa-miR-30d-5p** |
| **MKRN3** | **makorin ring finger protein 3** | **-0.96** | **hsa-miR-30d-5p** |
| **PDCD2L** | **programmed cell death 2-like** | **-0.94** | **hsa-miR-30d-5p** |
| **FGL2** | **fibrinogen-like 2** | **-0.93** | **hsa-miR-93-5p** |
| **CTDSPL** | **CTD (carboxy-terminal domain, RNA polymerase II, polypeptide A) small phosphatase-like** | **-0.92** | **hsa-miR-93-5p** |
| **EED** | **embryonic ectoderm development** | **-0.91** | **hsa-miR-30d-5p** |
| **GAN** | **gigaxonin** | **-0.9** | **hsa-miR-30d-5p** |
| **MEIOB** | **meiosis specific with OB domains** | **-0.86** | **hsa-miR-30d-5p** |
| **GPR137C** | **G protein-coupled receptor 137C** | **-0.84** | **hsa-miR-93-5p** |
| **ZBTB7A** | **zinc finger and BTB domain containing 7A** | **-0.84** | **hsa-miR-93-5p** |
| **CNRIP1** | **cannabinoid receptor interacting protein 1** | **-0.83** | **hsa-miR-30d-5p** |
| **SERF1A** | **small EDRK-rich factor 1A (telomeric)** | **-0.83** | **hsa-miR-93-5p** |
| **SERF1B** | **small EDRK-rich factor 1B (centromeric)** | **-0.83** | **hsa-miR-93-5p** |
| **B3GNT5** | **UDP-GlcNAc:betaGal beta-1,3-N-acetylglucosaminyltransferase 5** | **-0.82** | **hsa-miR-30d-5p** |
| **DLGAP1** | **discs, large (Drosophila) homolog-associated protein 1** | **-0.82** | **hsa-miR-30d-5p** |
| **KRBOX4** | **KRAB box domain containing 4** | **-0.82** | **hsa-miR-181b-5p** |
| **ATG12** | **autophagy related 12** | **-0.8** | **hsa-miR-30d-5p** |
| **HTR1F** | **5-hydroxytryptamine (serotonin) receptor 1F, G protein-coupled** | **-0.8** | **hsa-miR-30d-5p** |
| **DYNC1LI2** | **dynein, cytoplasmic 1, light intermediate chain 2** | **-0.79** | **hsa-miR-93-5p** |
| **HN1** | **hematological and neurological expressed 1** | **-0.79** | **hsa-miR-93-5p** |
| **MAP3K2** | **mitogen-activated protein kinase kinase kinase 2** | **-0.79** | **hsa-miR-93-5p** |
| **NAGK** | **N-acetylglucosamine kinase** | **-0.78** | **hsa-miR-93-5p** |
| **ENPP5** | **ectonucleotide pyrophosphatase/phosphodiesterase 5 (putative)** | **-0.77** | **hsa-miR-93-5p** |
|  |  |  |  |
|  | | | |

**Note: TargetScanHuman 7.2, http://www.targetscan.org/vert_72/**
