# Supplementary figures and images for: MicroRNA signature constituted of miR-30d, miR-93, and miR-181b is a promising prognostic marker in primary central nervous system lymphoma
Source: PLoS One. 2019 Jan 7;14(1):e0210400. doi: 10.1371/journal.pone.0210400 (PMC6322780; doi:10.1371/journal.pone.0210400)

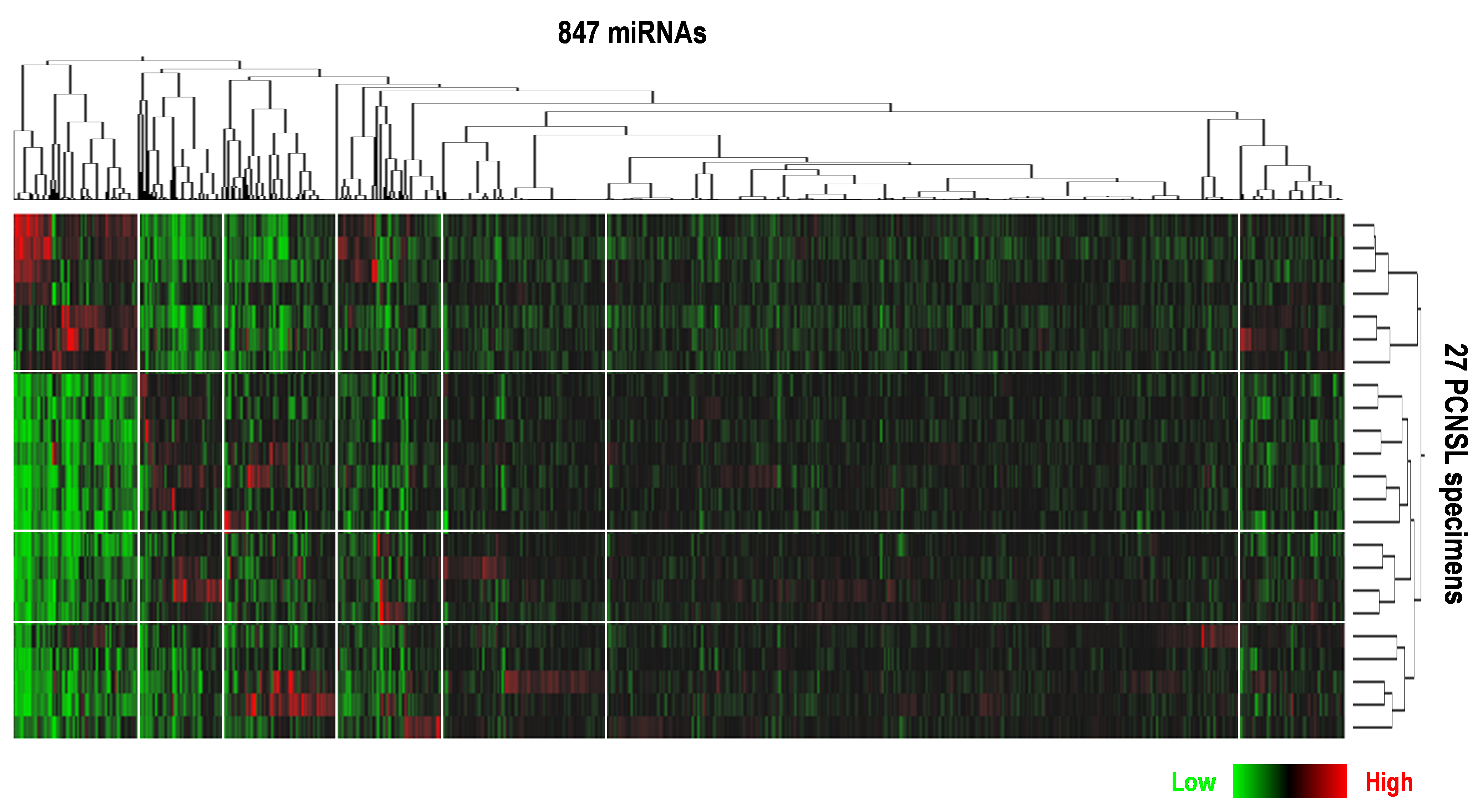

Supplement: S1 Fig — Hierarchical clustering analysis was performed. Red and green indicate high and low expression, respectively. (TIF) [file pone.0210400.s002.tif]

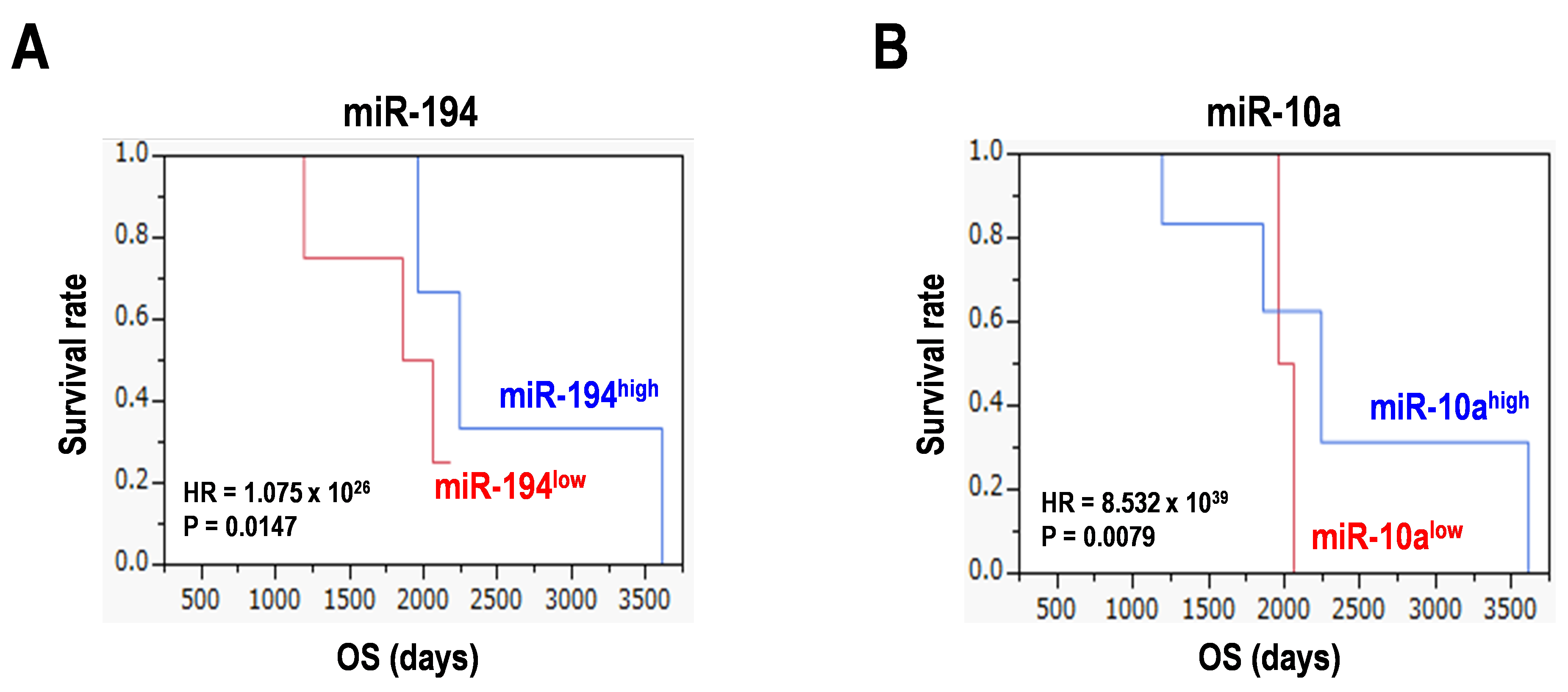

Supplement: S2 Fig — The patients were divided by median expression values of (A) miR-194 and (B) miR-10a. Kaplan-Meier analyses were performed. OS, overall survival (days). (TIF) [file pone.0210400.s003.tif]

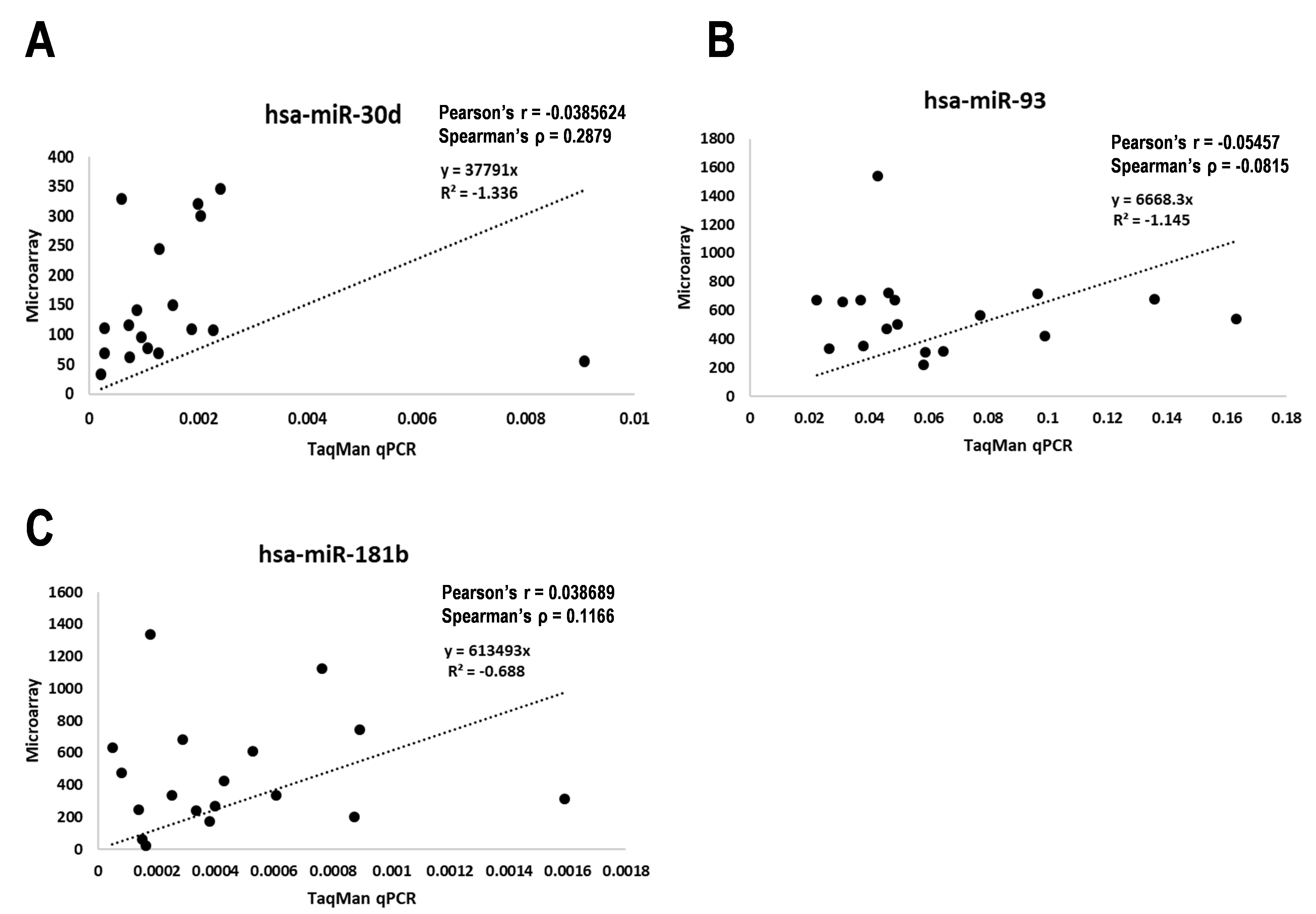

Supplement: S3 Fig — (A) miR-30d, (B) miR-93, and (C) miR-181b. Scatter plots were shown with statistic results. Each dot represents a PCNSL specimen. (TIF) [file pone.0210400.s004.tif]
